# Synthesis of diverse dihydropyrimidine-related scaffolds by fluorous benzaldehyde-based Biginelli reaction and post-condensation modifications

**DOI:** 10.3762/bjoc.7.150

**Published:** 2011-09-16

**Authors:** Bruno Piqani, Wei Zhang

**Affiliations:** 1 Department of Chemistry, University of Massachusetts Boston, 100 Morrissey Boulevard, Boston, MA 02125, USA

**Keywords:** Biginelli reaction, dihydropyrimidine, diversity-oriented synthesis, fluorous, Suzuki coupling

## Abstract

Dihydropyrimidinones and dihydropyrimidinethiones generated from the Biginelli reactions of perfluorooctanesulfonyl-attached benzaldehydes are used as common intermediates for post-condensation modifications such as cycloaddition, Liebeskind–Srogl reaction and Suzuki coupling to form biaryl-substituted dihydropyrimidinone, dihydropyrimidine, and thiazolopyrimidine compounds. The high efficiency of the diversity-oriented synthesis is achieved by conducting a multicomponent reaction for improved atom economy, under microwave heating for fast reaction, and with fluorous solid-phase extractions (F-SPE) for ease of purification.

## Introduction

**Scheme 1:**
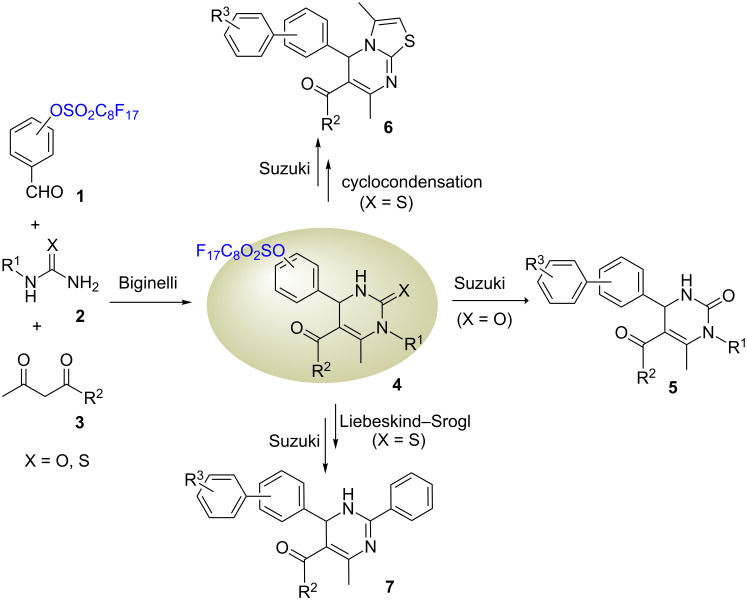
Synthesis of diverse dihydropyrimidine-related compounds.

Dihydropyrimidinone and dihydropyrimidine derivatives have broad biologically activities. Many synthetic samples have been studied as antibacterial, antiviral, antihypertensive, and anticancer agents [1], and the natural products containing these heterocyclic moieties have been studied as new leads for AIDS therapies [2]. The Biginelli reaction of a β*-*keto ester, an aldehyde, and urea is considered as one of the most efficient ways to synthesize dihydropyrimidinones [3]. This acid-catalyzed reaction can be conducted under conventional or microwave heating [4–5]. Reported in this paper is a diversity-oriented synthesis of biaryl-substituted dihydropyrimidinone **5**, thiazolopyrimidine **6**, and dihydropyrimidine **7** compounds (Scheme 1). The perfluorooctanesulfonyl-attached benzaldehydes **1** were used as a key component for the Biginelli reactions [6]. The Biginelli products **4** were used as a common intermediate for post-condensation reactions including cycloaddition, Liebeskind–Srogl reaction and Suzuki coupling to form three different heterocyclic skeletons. The high efficiency of the diversity-oriented synthesis was achieved by conducting fast, microwave-heated reactions and simple fluorous solid-phase extractions (F-SPE) for purification [7]. The perfluorooctanesulfonyl group served as a phase tag for F-SPE and also as a convertible linker for the Suzuki coupling to introduce biaryl functionality to the heterocyclic skeletons [8–12].

## Result and Discussion

Fluorous benzaldehydes **1** were prepared by the reaction of phenols with perfluorooctanesulfonyl fluoride, by following the reported procedure [13]. Compounds **1** were used as a limiting agent to react with urea/thiourea **2** and acetylacetone **3** for the Biginelli reactions. The reactions were promoted by Yb(OTf)_3_ as a catalyst [14–15], acetonitrile as a solvent, and under microwave irradiation at 120 °C for 20 min. This optimized condition was developed after other solvents, including water, EtOH and toluene, and different microwave reaction temperatures (100–130 °C) and times (10–20 min) were explored. The Biginelli products were separated from the reaction mixtures by F-SPE eluted with fluorophobic 80:20 MeOH/H_2_O and then fluorophilic 100% MeOH or acetone [7]. The fluorous Biginelli products were collected from the MeOH fraction to give dihydropyrimidinones **4a–d** and dihydropyrimidinethiones **4e**,**f** in 85–95% yields (Table 1). The Biginelli products **4a–e** were used for Suzuki coupling reactions to remove the fluorous linker and introduce the biaryl functional group. The coupling reactions were promoted by microwave heating at 140 °C for 30 min with Pd(pddf)Cl_2_ as a catalyst, Cs_2_CO_3_ as a base, and 4:4:1 acetone/toluene/H_2_O as a solvent [13]. Dihydropyrimidinones **4a–d** gave the expected products **5a–h** in 51–68% yield after F-SPE and flash chromatography purification. However, no reactions occurred with the dihydropyrimidinethiones **4e**,**f** under these reaction conditions.

**Table 1:** Biginelli reactions followed by Suzuki reactions of dihydropyrimidinones and dihydropyrimidinethiones.

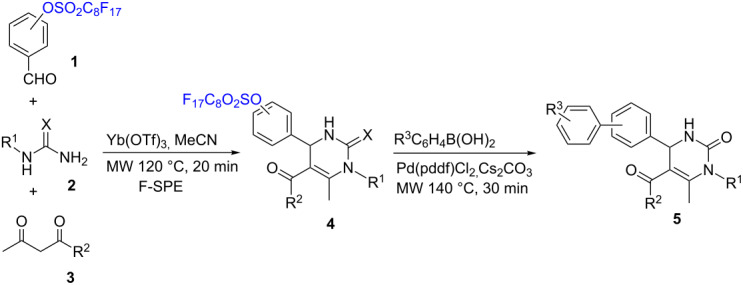						

R^1^	R^2^	X	F-Sulfonyl position	**4** (yield)	R^3^	**5** (yield)

CH_3_	CH_3_	O	*meta*	**4a** (91%)	*p*-OCH_3_	**5a** (67%)
					H	**5b** (56%)
CH_3_	OCH_3_	O	*meta*	**4b** (95%)	*p*-OCH_3_	**5c** (57%)
					H	**5d** (51%)
CH_3_	CH_3_	O	*para*	**4c** (90%)	*p*-OCH_3_	**5e** (68%)
					H	**5f** (62%)
CH_3_	OCH_3_	O	*para*	**4d** (88%)	*p*-OCH_3_	**5g** (58%)
					H	**5h** (60%)
H	CH_3_	S	*meta*	**4e** (89%)	H	-
H	OCH_3_	S	*meta*	**4f** (85%)	H	-

Since dihydropyrimidinethiones **4e**,**f** failed to give Suzuki coupling products, our next effort was to convert them to thiazolopyrimidine through cyclocondensation with chloroacetone [16–17]. The reaction was performed in water under microwave heating at 120 °C for 30 min to afford thiazolopyrimidines **8a** and **8b** in 89% and 85% yields, respectively, after F-SPE. Suzuki reactions of **8a** and **8b** with four boronic acids yielded 5-biaryl-5*H*-thiazolo[3,2-a]pyrimidines **6a–h** in 55–64% yields after F-SPE and flash chromatography purifications (Table 2).

**Table 2:** Synthesis of biaryl-substituted thiazolopyrimidines.

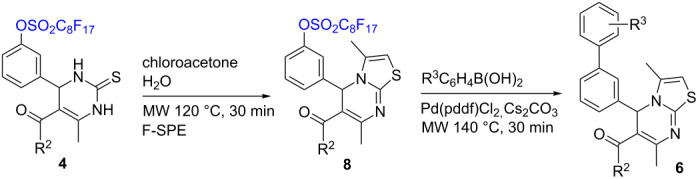				

**4**	R^2^	**8**	R^3^	**6** (yield)

**4e**	CH_3_	**8a** (89%)	H	**6a** (61%)
			*p*-OCH_3_	**6b** (64%)
			*m*-Cl	**6c** (56%)
			*p*-CH_3_	**6d** (62%)
**4f**	OCH_3_	**8b** (85%)	H	**6e** (58%)
			*p*-OCH_3_	**6f** (55%)
			*m*-Cl	**6g** (63%)
			*p*-CH_3_	**6h** (55%)

Dihydropyrimidinethione **4f** was used for the Liebeskind–Srogl coupling reaction with a phenylboronic acid to convert to 2-aryl-1,6-dihydropyrimidine **9** [18–20]. The reaction was performed following a literature procedure [21] and was catalyzed by Pd(PPh_3_)_4_ and copper(I) thiophene-2-carboxylate (CuTC) under microwave heating at 100 °C for 25 min to afford aryl-substituted dihydropyrimidine **9** in 76% yield. This compound was then subjected to Suzuki coupling reactions with four boronic acids to yield 2-aryl-6-biaryl substituted dihydropyrimidines **7a–d** after F-SPE and flash chromatography purifications (Table 3).

**Table 3:** Synthesis of 2-aryl-6-biaryl-substituted dihydropyrimidines.

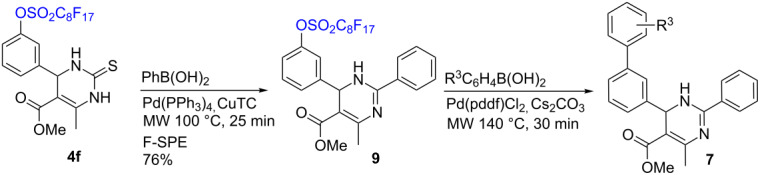	

R^3^	**7** (yield)

H	**7a** (45%)
*p*-OCH_3_	**7b** (48%)
*m*-Cl	**7c** (31%)
*p*-CH_3_	**7d** (48%)

## Conclusion

We have developed a new application of perfluorooctanesulfonyl-attached benzaldehydes for the diversity-oriented synthesis of heterocyclic scaffolds. The intermediates obtained from the Biginelli reaction were used for post-condensation modifications to afford biaryl-substituted dihydropyrimidinone, dihydropyrimidine, and thiazolopyrimidine compounds. A set of reaction and separation techniques such as multicomponent reactions, microwave heating, and F-SPE was employed to increase the synthetic efficiency. The fluorous sulfonyl group not only served as a phase tag for F-SPE separation, but also as a cleavable linker for the Suzuki coupling reactions.

## Experimental

Typical Biginelli reaction procedure: Synthesis of 5-acetyl-4-(4- (perfluorooctylsulfonyloxy)phenyl)-1,6-dimethyl-3,4-dihydropyrimidin-2(1*H*)-one (**4c**)

A solution of *p-*perfluorooctanesulfonyl benzaldehyde **1** (1.2 g, 2.0 mmol), methylurea **2** (0.18 g, 2.4 mmol), methyl acetoacetate **3** (0.35 g, 3.0 mmol) and Yb(OTf)_3_ (124 mg, 0.2 mmol) in 2 mL of acetonitrile was heated in a Biotage Initiator microwave synthesizer at 120 °C for 20 min. The resulting mixture was purified by F-SPE eluted with 40 mL of 80:20 MeOH/H_2_O and then 40 mL of acetone. The acetone fraction was concentrated to give **4c** (1.3 g) in 90% yield.

### 

#### Typical Suzuki reaction procedure: Synthesis of 5-acetyl-4-(4'-methoxy-[1,1'-biphenyl]-3-yl)-1,6-dimethyl-3,4-dihydropyrimidin-2(1*H*)-one (**5a**)

A solution of **4a** (75 mg, 0.1 mmol), 4-methoxyphenylboronic acid (23 mg, 0.15 mmol), Cs_2_CO_3_ (81 mg, 0.25 mmol) and Pd(dppf)Cl_2_ (16 mg, 0.02 mmol) in 3 mL of 4:1:4 acetone/H_2_O/toluene was heated in a Biotage Initiator microwave synthesizer at 140 °C for 30 min. The resulting mixture was purified by flash chromatography to give **5a** (24 mg) in 67% yield.

#### Typical procedure for cyclocondensation of **4e,f**. Synthesis of methyl 3,7-dimethyl-5-(3-(perfluorooctylsulfonyloxy)phenyl)-5*H*-thiazolo[3,2-a]pyrimidine-6-carboxylate (**8b**)

A solution of 3,4-dihydropyrimidinethione **4f** (0.76 g, 1 mmol), chloroacetone (185 mg, 1.5 mmol) in 2 mL water was heated in Biotage Initiator microwave synthesizer at 120 °C for 30 min. The resulting mixture was purified by F-SPE eluted with 30 mL of 80:20 MeOH/H_2_O and then 30 mL of acetone. The acetone fraction was concentrated to give **8b** (0.67 g) in 85% yield.

#### Typical Liebeskind**–**Srogl reaction procedure. Synthesis of methyl 4-methyl-6-(3-(perfluorooctylsulfonyloxy)phenyl)-2-phenyl-1,6-dihydropyrimidine-5-carboxylate (**9**)

A solution of 3,4-dihydropyrimidinethione **4f** (152 mg, 0.20 mmol), phenylboronic acid (82 mg, 0.3 mmol), CuTC (95 mg, 0.6 mmol), and Pd(PPh_3_)_4_ (3 mol %) in 2 mL THF was heated in Biotage Initiator microwave synthesizer at 100 °C for 25 min. The mixture was purified by F-SPE eluted with 30 mL of 80:20 MeOH/H_2_O and then 30 mL of acetone. The acetone fraction was concentrated to give **9** (0.85 g) in 76% yield.

## Supporting Information

File 1 LC–MS, ^1^H NMR and ^13^C NMR data and spectra for compounds **4c, 5a, 6b, 7b, 8b, 9**.
