# Supplementary material for: Synthesis of diverse dihydropyrimidine-related scaffolds by fluorous benzaldehyde-based Biginelli reaction and post-condensation modifications
Source: Beilstein J Org Chem. 2011 Sep 16;7:1294–8. doi: 10.3762/bjoc.7.150 (PMC3182439; doi:10.3762/bjoc.7.150)

Supporting Information
for

Synthesis of diverse dihydropyrimidine-related scaffolds by fluorous benzaldehyde-based Biginelli reaction and post-condensation modifications

Bruno Piqani and Wei Zhang*

Address: Department of Chemistry, University of Massachusetts Boston, 100 Morrissey Boulevard, Boston, MA 02125, USA

Email: Bruno Piqani - piqani@gmail.com; Wei Zhang* - wei2.zhang@umb.edu

*Corresponding author

LC–MS, ^{1}H NMR and ^{13}C NMR data and spectra for compounds
4c, 5a, 6b, 7b, 8b, 9

General methods:

The ^{1}H NMR and ^{13}C NMR spectra were recorded on a 300 MHz Varian NMR spectrometer, in $CDCl_3$ solvent with tetramethylsilane as the internal standard. The temperature was 25 °C (accuracy ±1 °C). Splitting patterns of ^{1}H NMR spectra are designated as s, singlet; d, doublet; t, triplet; q, quartet; and m, multiplet. LC–MS spectra were recorded on an Agilent 2100 system. A C18 column (5.0 µm, 6.0 × 50 mm) was used for the separation. The mobile phases were methanol and water, both containing 0.05% formic acid. A linear gradient was used to increase the ratio from 25:75 v/v methanol/water to 100% methanol over 5.0 min at a flow rate of 0.7 mL/min. The UV measurements were made at 210 nm and 254.4 nm. Mass spectra were recorded by atmospheric-pressure chemical ionization. All reactions were carried out in a self-tuning single mode Biotage Initiator Microwave Synthesizer. Purification of intermediates took place in a Thermo Scientific 16 SPE Vacuum Manifold.

General procedure for the preparation of dihydropyrimidinones and dihydropyrimidinethiones derivatives and spectral data for selected compounds.

A general procedure for the synthesis of dihydropyrimidinone compound 4c. A solution of *p*-perfluorooctanesulfonyl benzaldehyde **1** (1.2 g, 2.0 mmol), methylurea **2** (0.18 g, 2.4 mmol), methyl acetoacetate **3** (0.35 g, 3.0 mmol) and $Yb(OTf)_3$ (124 mg, 0.2 mmol) in acetonitrile (2 mL) in a microwave reaction tube was heated in Biotage microwave reactor at 120 °C for 20 min. The resulting mixture was purified by F-SPE eluted with 40 mL of 80:20 $MeOH/H_2O$ and then 40 mL of acetone. The acetone (fluorous) fraction was concentrated to give **4c** (1.3 g, 90% yield).

5-Acetyl-4-(4-(perfluorooctylsulfonyloxy)phenyl)-1,6-dimethyl-3,4-dihydropyrimidin-2(1*H*)-one (4c):

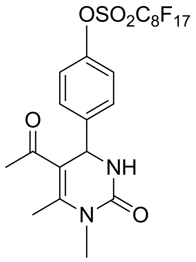

LC–MS ($APCI^+$) *m/z* 743 $[M + 1]^+$; ^1H NMR (300 MHz, $CDCl_3$) δ 7.34 (d, *J* = 8.1 Hz, 2H), 7.23 (d, *J* = 7.7 Hz, 2H), 6.27 (d, *J* = 3.0 Hz, 1H), 5.45 (d, *J* = 3.0 Hz, 1H), 3.23 (s, 3H), 2.47 (s, 3H), 2.24 (s, 3H); ^{13}C NMR (75.5 MHz, $CDCl_3$) δ 195.8, 153.8, 149.3, 148.8, 142.8, 133.6 128.2, 121.9, 121.0 120.3, 119.9, 118.6, 117.4 116.6, 114.8, 113.5, 53.2, 30.6, 29.7, 17.2; HRMS (ES^+): *m/z* $[M + H]^+$ calcd for $C_{22}H_{15}F_{17}N_2O_5S$: 743.0508; found: 743.0514.

A general procedure for Suzuki reactions. Synthesis of compounds 5a. A solution of **4a** (75 mg, 0.1 mmol), 4-methoxyphenylboronic acid **10** (23 mg, 0.15 mmol), Cs_2CO_3 (81 mg, 0.25 mmol) and $Pd(dppf)Cl_2$ (16 mg, 0.02 mmol) in 4:1:4 acetone:H_2O:toluene (3 mL) was heated under microwave irradiation at 140 °C for 30 min. The resulting mixture was purified by flash chromatography with 0–50% gradient of EtOAc/hexanes to give **5a** (24 mg, 67% yield).

Methyl 4-(4'-methoxy-[1,1'-biphenyl]-3-yl)-1,6-dimethyl-2-oxo-1,2,3,4-tetrahydropyrimidine-5-carboxylate (5a)

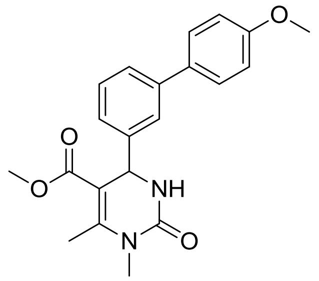

LC–MS (APCI$^+$) *m/z* 367 [M + 1]$^+$; ^{1}H NMR (300 MHz, $CDCl_3$) δ 7.57 (d, *J* = 11.7 Hz, 1H), 7.49–7.42 (m, 3H), 7.34 (t, *J* = 15.3 Hz, 1H), 7.19–6.95 (m, 2H), 6.76 (s, 1H), 6.03 (d, *J* = 3.0 Hz, 1H), 5.45 (d, *J* = 3.0 Hz, 1H), 3.85 (s, 3H), 3.67 (s, 3H), 3.24 (s, 3H), 2.53 (s, 3H); ^{13}C NMR (75.5 MHz, $CDCl_3$) δ 166.5, 154.2, 149.7, 142.1, 140.6, 140.5 128.8, 128.7, 127.4, 127.3, 126.9, 126.5, 103.9, 54.9, 54.3, 53.2, 30.4, 16.6; HRMS (ES$^+$): *m/z* [M + H]$^+$ calcd for $C_{21}H_{22}N_2O_4$: 367.1658; found: 367.1659.

A general procedure for the synthesis of compounds 8b. A solution of 3,4-dihydropyrimidine-thione **4f** (0.76 g, 1 mmol), chloroacetone (185 mg, 1.5 mmol) in water (2 mL) was heated under microwave at 120 °C for 30 min. The resulting mixture was purified by F-SPE eluted with 30 mL of 80:20 MeOH/H_2O and then 30 mL of acetone. The Acetone (fluorous) fraction was concentrated to give **8b** (0.67 g, 85% yield).

Methyl 3,7-dimethyl-5-(3-(perfluorooctylsulfonyloxy)phenyl)-5*H*-thiazolo[3,2-a]pyrimidine-6-carboxylate (8b)

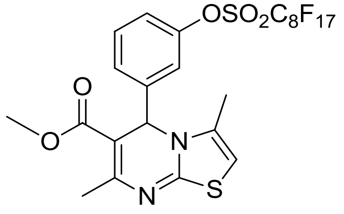

LC–MS (APCI$^+$) *m/z* 799 [M + 1]$^+$; ^{1}H NMR (300 MHz, $CDCl_3$) δ 7.85 (d, *J* = 7.5Hz, 2H) 7.74 (d, *J* = 2.7 Hz, 2H), 7.43–7.32 (m, 3H), 7.27–7.18 (m, 2H), 6.22 (s, 1H), 6.06 (s, 1H), 3.85 (s, 3H), 2.40 (s, 3H), 2.07(s, 3H); ^{13}C NMR (75.5 MHz, $CDCl_3$) δ 169.7, 165.5, 153.8, 152.7, 149.6, 143.2 135.0, 131.2, 128.5, 126.3, 121.6, 120.5, 119.5, 118.9, 118.3, 117.7, 117.0 116.2, 114.4, 113.7, 57.2, 51.1, 23.1, 13.8.

Methyl-5-(4'-methoxy-[1,1'-biphenyl]-3-yl)-3,7-dimethyl-5*H*-thiazolo[3,2-a]pyrimidine-6-carboxylate (6b).

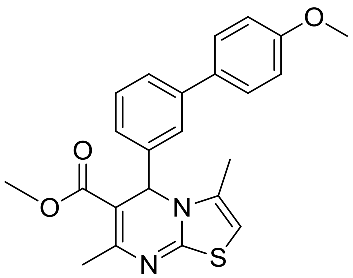

LC–MS (APCI$^+$) *m/z* 407 $[M + 1]^+$; ^{1}H NMR (300 MHz, $CDCl_3$) δ 7.49–7.41 (m, 3H), 7.32–6.09 (m, 3H), 6.96 (d, *J* = 10.0 Hz, 2H), 6.68 (s, 1H), 6.16 (s, 1H), 3.84 (s, 3H), 2.40 (s, 6H), 2.12 (s, 3H); ^{13}C NMR (75.474 MHz, $CDCl_3$) δ 169.8, 166.5, 153.8, 152.7, 147.8, 141.1 136.4, 132.9, 129.2, 128.1, 126.4 124.6, 114.2, 57.2, 55.3, 51.3, 24.5, 13.9; HRMS (ES$^+$): *m/z* $[M + H]^+$ calcd for $C_{23}H_{22}N_2O_3S$: 407.1426; found: 407.1429.

A general procedure for the synthesis of compound 9. A solution of 3,4-dihydropyrimidine-thione **4f** (152 mg, 0.20 mmol), phenylboronic acid **10** (82 mg, 0.3 mmol), CuTC (95 mg, 0.6 mmol), and $Pd(PPh_3)_4$ (3mol %) in THF (2 mL) was heated under microwave at 100 °C for 25 min. The mixture was purified by F-SPE eluted with 30 mL of 80:20 MeOH/H_2O and then 30 mL of acetone. The acetone (fluorous) fraction was concentrated to give **9** (0.85 g, 76 % yield).

Methyl 4-methyl-6-(3-(perfluorooctylsulfonyloxy)phenyl)-2-phenyl-1,6-dihydropyrimidine-5-carboxylate (9).

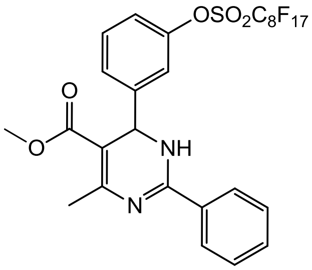

LC–MS (APCI$^+$) *m/z* 805 [M + 1]$^+$; ^{1}H NMR (300 MHz, $CDCl_3$) δ 7.73–7.23 (m, 3H), 7.19–7.04 (m, 2H), 5.62 (s, 1H), 3.58 (s, 3H), 3.72 (s, 3H), 2.41 (s, 3H); ^{13}C NMR (75.5 MHz, $CDCl_3$) δ 170.1, 166.9, 160.2, 153.0, 149.9 146.0, 133.6, 132.3, 130.4, 128.9, 127.8, 127.3, 127.0, 120.5, 120.2, 119.7, 118.6, 117.6, 116.4, 114.9, 55.9, 51.4, 29.7, 23.8; HRMS (ES$^+$): *m/z* [M + H]$^+$ calcd for $C_{27}H_{17}F_{17}N_2O_5S$: 805.0665; found: 805.0670.

Methyl-6-(4'-methoxy-[1,1'-biphenyl]-3-yl)-2-phenyl-1,6-dihydropyrimidine-5-carboxylate (7b).

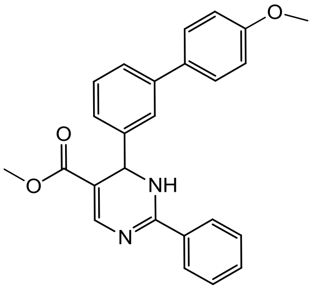

LC–MS (APCI$^+$) *m/z* 413 [M + 1]$^+$; ^{1}H NMR (300 MHz, $CDCl_3$) δ 7.99 (d, *J* = 8.1 Hz, 2H), 7.68 (s, 1H), 7.56–7.48 (m, 3H), 7.40–7.26 (m, 3H), 6.74 (d, *J* = 8.1 Hz, 2H), 5.79 (s, 1H), 3.64 (s, 6H), 2.65 (s, 3H); ^{13}C NMR (75.5 MHz, $CDCl_3$) δ 170.2, 166.7, 153.8, 152.7, 149.6, 141.1 136.4, 133.0 132.3, 130.2, 129.4, 129.2, 128.4, 126.7, 124.6, 114.2, 57.2, 55.9, 51.7, 23.5; HRMS (ES$^+$): *m/z* [M + H]$^+$ calcd for $C_{21}H_{24}N_4O_5$: 413.1865; found: 413.1864.

ıple Name : A2B3C6

===

[. Operator : Bruno Seq. Line : 1

[. Instrument : Instrument 1 Location : Vial 1

ection Date : 5/11/2011 7:41:29 AM Inj : 1

Inj Volume : 4 µl

:hod : C:\CHEM32\1\METHODS\GEMETHOD1.M

;t changed : 4/27/2011 11:04:42 AM by J

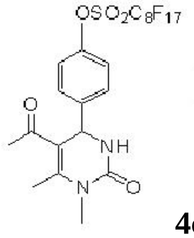

4c

Current Chromatogram(s)

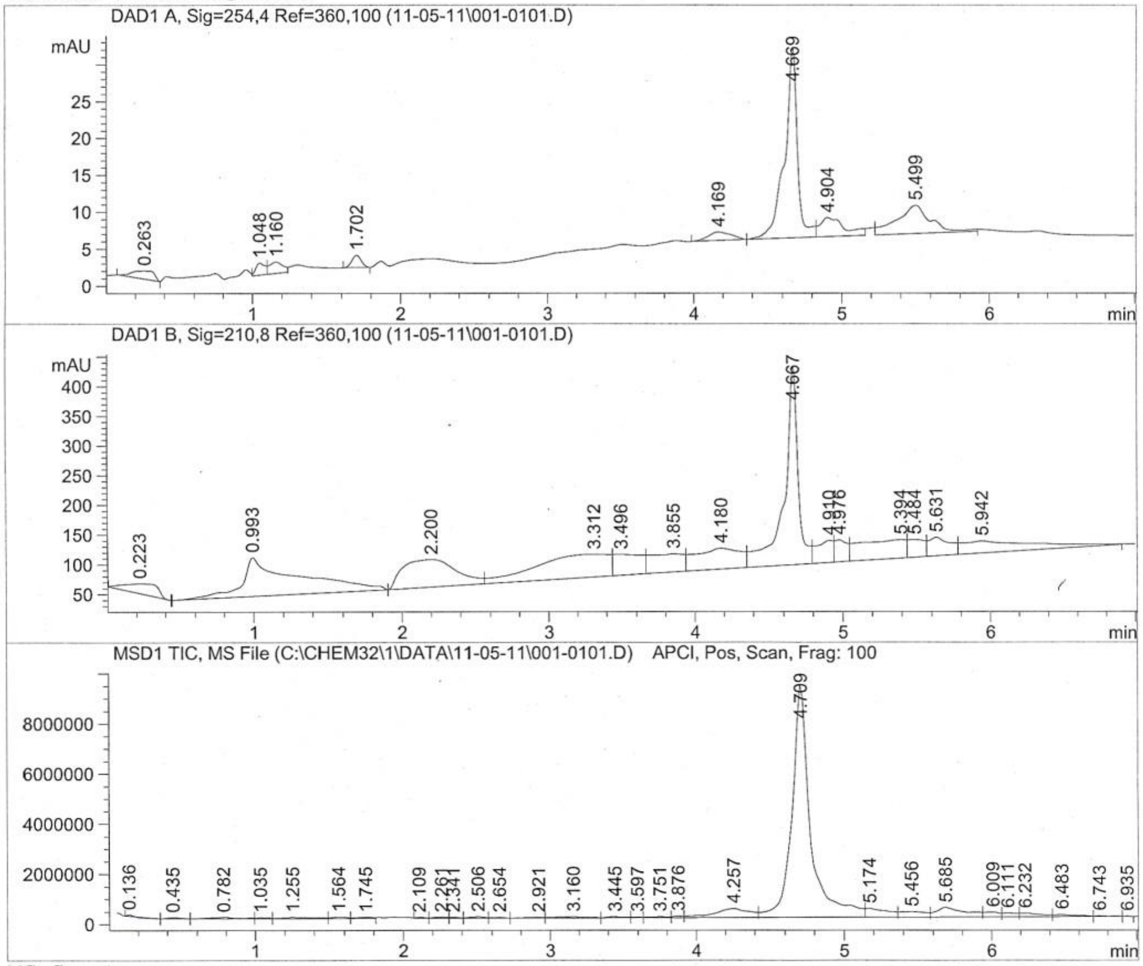

MS Spectrum

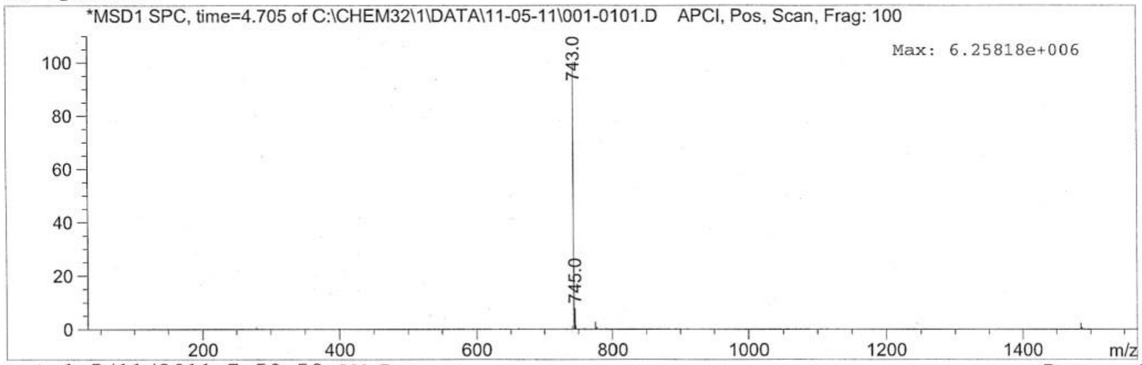

;trument 1 5/11/2011 7:50:50 AM Bruno Page 1 of

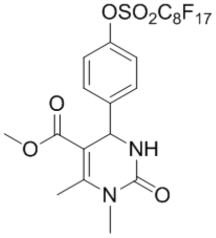

4c

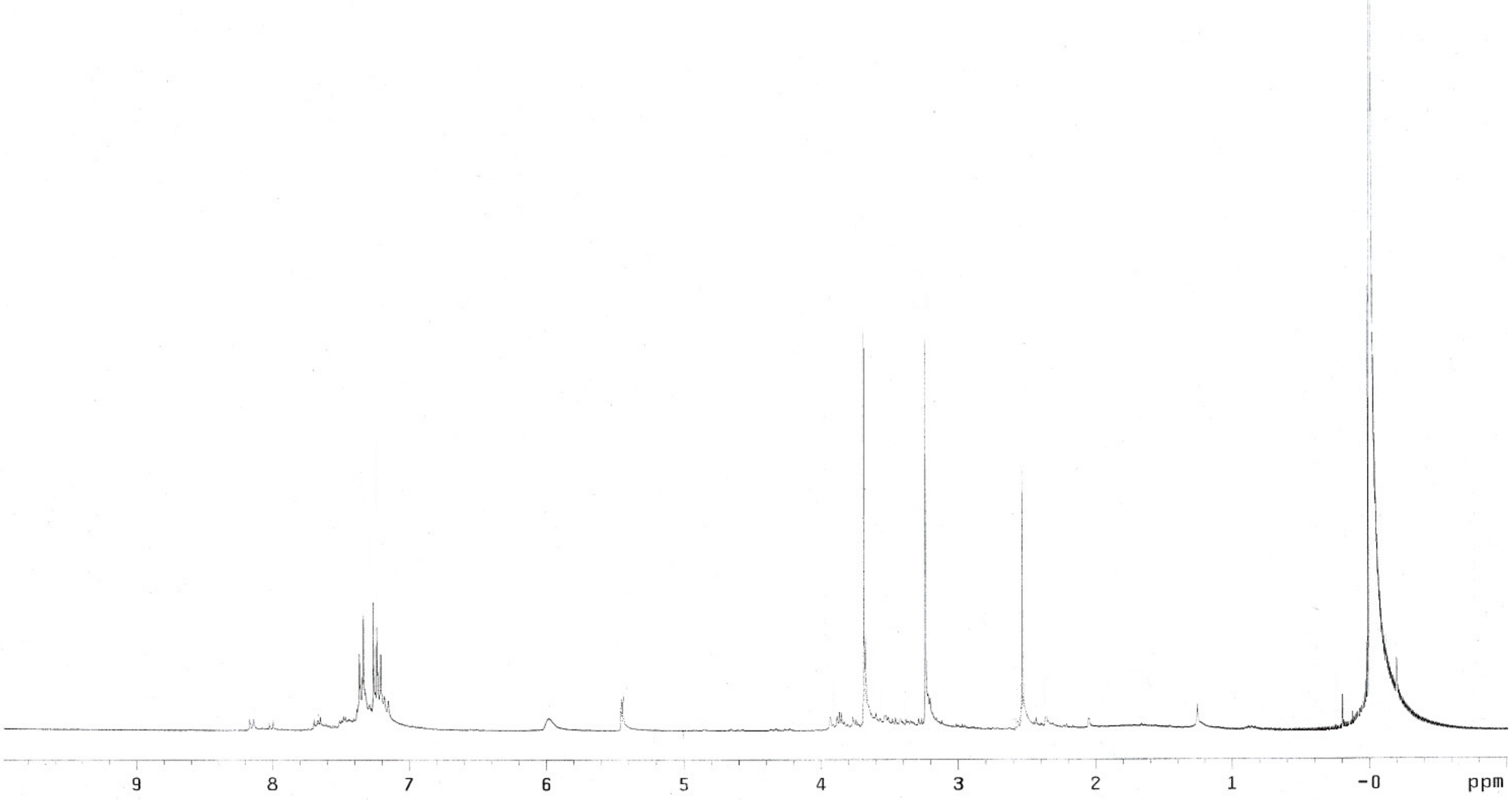
STANDARD 1H OBSERVE
Pulse Sequence: s2pul
Solvent: CDCl3
Ambient temperature
File: A2B3C5
GEMINI-300BB "Mighty300"
Relax. delay 1.000 sec
Pulse 43.4 degrees
Acq. time 1.998 sec
Width 4500.5 Hz
256 repetitions
OBSERVE H1, 300.1266235 MHz
DATA PROCESSING
FT size 32768
Total time 13 min, 16 sec
9
8
7
6
5
4
3
2
1
-0
ppm

13C OBSERVE

Pulse Sequence: s2pul
Solvent: CDCl3
Ambient temperature
File: A2B3C6-C13
GEMINI-300BB "Mighty300"

Relax. delay 2.000 sec
Pulse 67.8 degrees
Acq. time 1.815 sec
Width 20000.0 Hz
1028 repetitions
OBSERVE C13, 75.4669013 MHz
DECOUPLE H1, 300.1281260 MHz
Power 36 dB
continuously on
WALTZ-16 modulated
DATA PROCESSING
Line broadening 1.0 Hz
FT size 131072
Total time 1 hr, 10 min, 46 sec

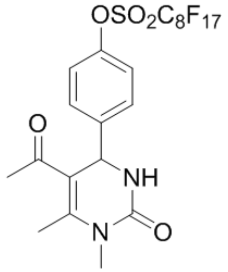

4c

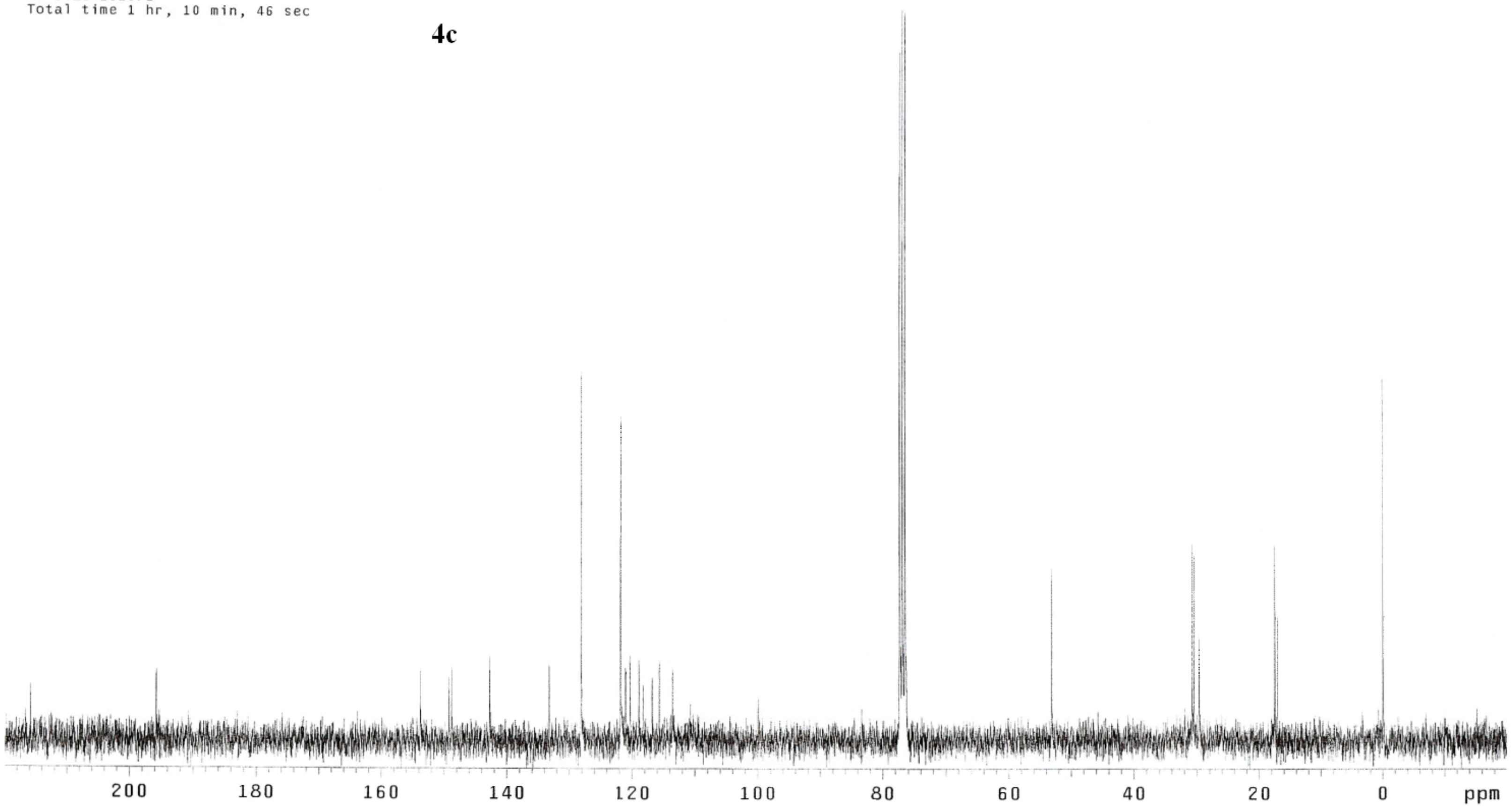

```
Print of all graphic windows
Data File   : C:\CHEM32\1\DATA\11-05-03\003-0301.D
Sample Name : A2B1B5D1
=====================================================================
Acq. Operator    : B                              Seq. Line :    3
Acq. Instrument  : Instrument 1                    Location : Vial 3
Injection Date   : 5/3/2011 11:30:47 AM                 Inj :    1
                                                 Inj Volume : 4 µl
Method           : C:\CHEM32\1\METHODS\GEMETHOD1.M
Last changed     : 4/27/2011 11:04:42 AM by J
```

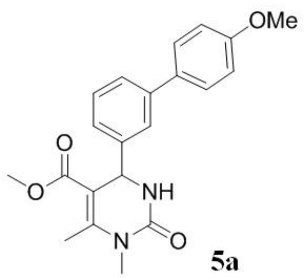

5a

Current Chromatogram(s)

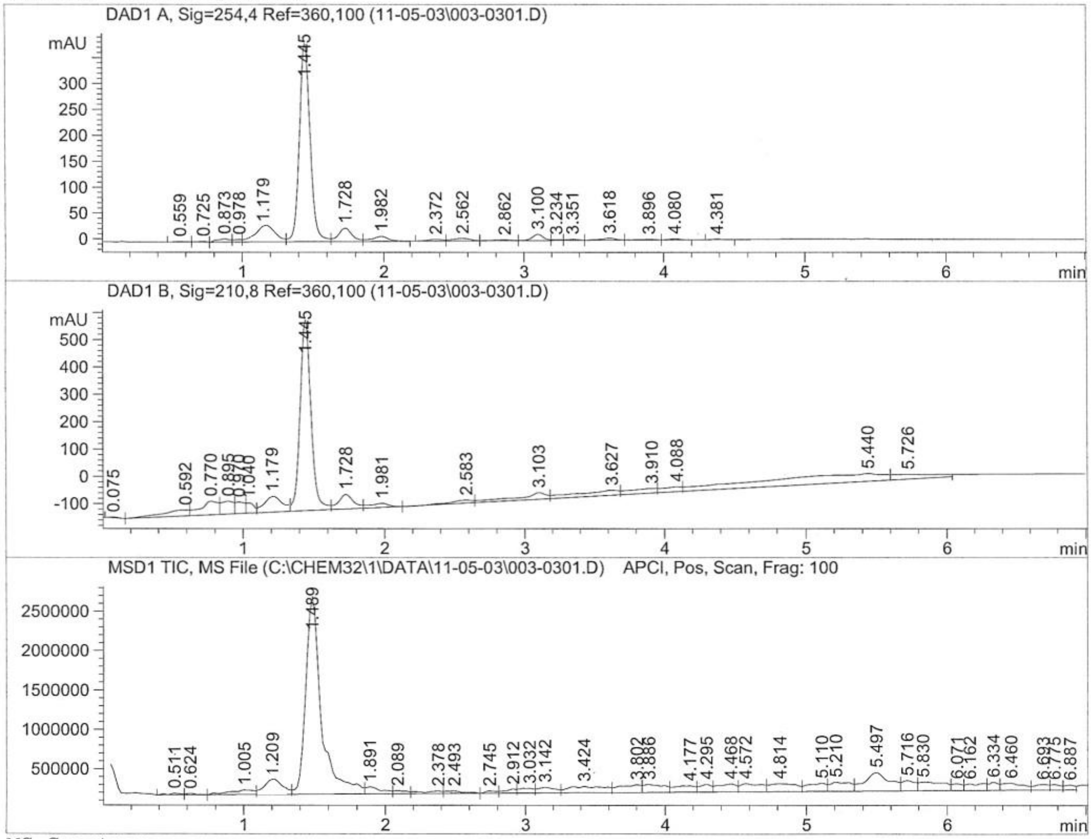

MS Spectrum

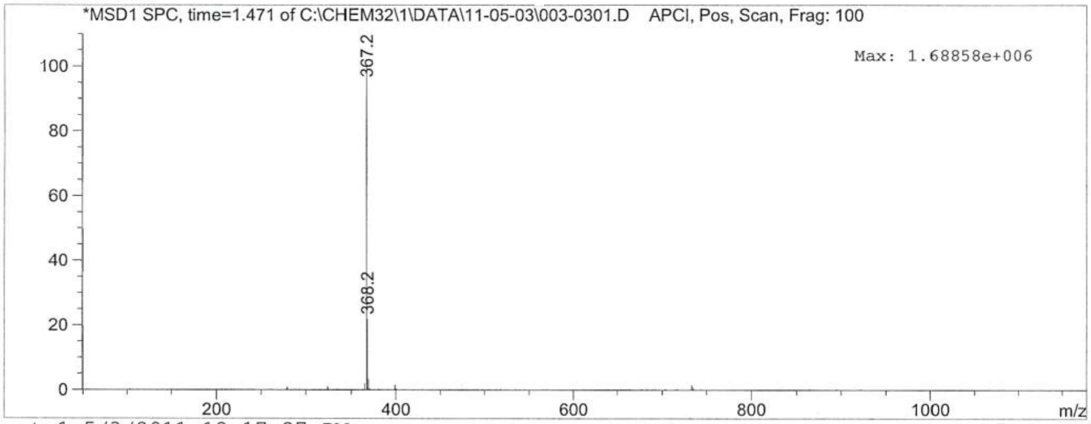

Instrument 1 5/3/2011 12:17:27 PM Page 1 of

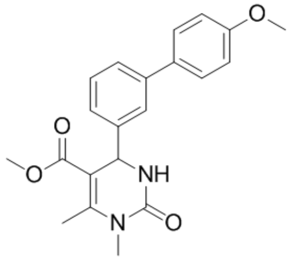

5a

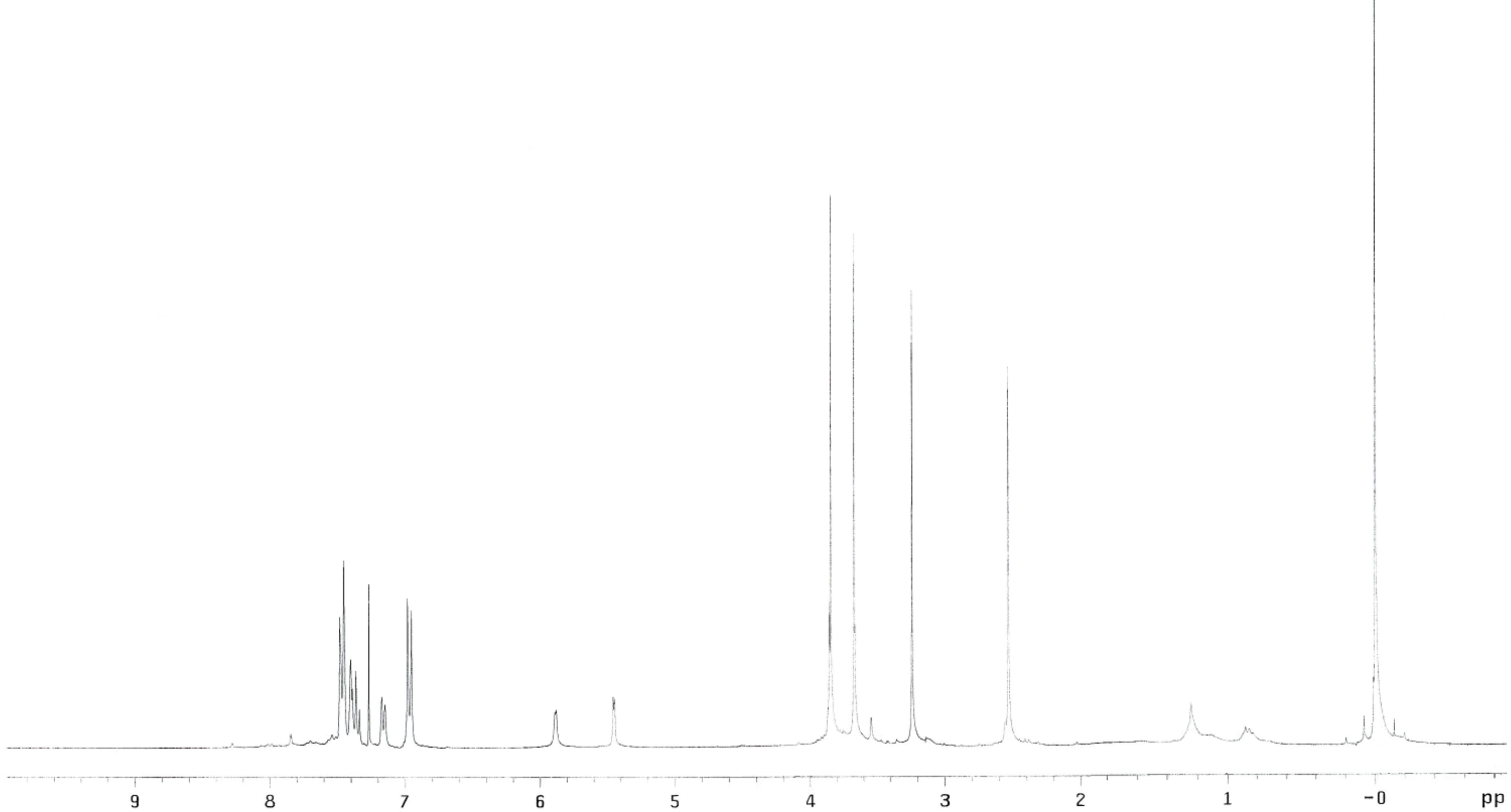
STANDARD 1H OBSERVE
Pulse Sequence: s2pul
Solvent: CDCl3
Ambient temperature
GEMINI-300BB "Mighty300"
Relax. delay 1.000 sec
Pulse 43.4 degrees
Acq. time 1.998 sec
Width 4500.5 Hz
64 repetitions
OBSERVE H1, 300.1266208 MHz
DATA PROCESSING
FT size 32768
Total time 0 min, 0 sec
9
8
7
6
5
4
3
2
1
-0
pp

13C OBSERVE

Pulse Sequence: s2pul
 Solvent: CDCl3
 Ambient temperature
GEMINI-300BB "Mighty300"

 Relax. delay 2.000 sec
 Pulse 67.8 degrees
 Acq. time 1.815 sec
 Width 20000.0 Hz
 1028 repetitions
OBSERVE C13, 75.4669007 MHz
DECOUPLE H1, 300.1281260 MHz
 Power 36 dB
 continuously on
 WALTZ-16 modulated
DATA PROCESSING
 Line broadening 1.0 Hz
FT size 131072
Total time 1 hr, 10 min, 46 sec

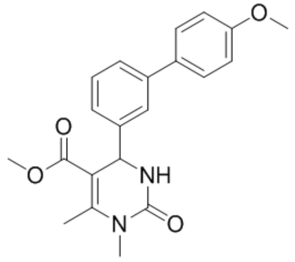

5a

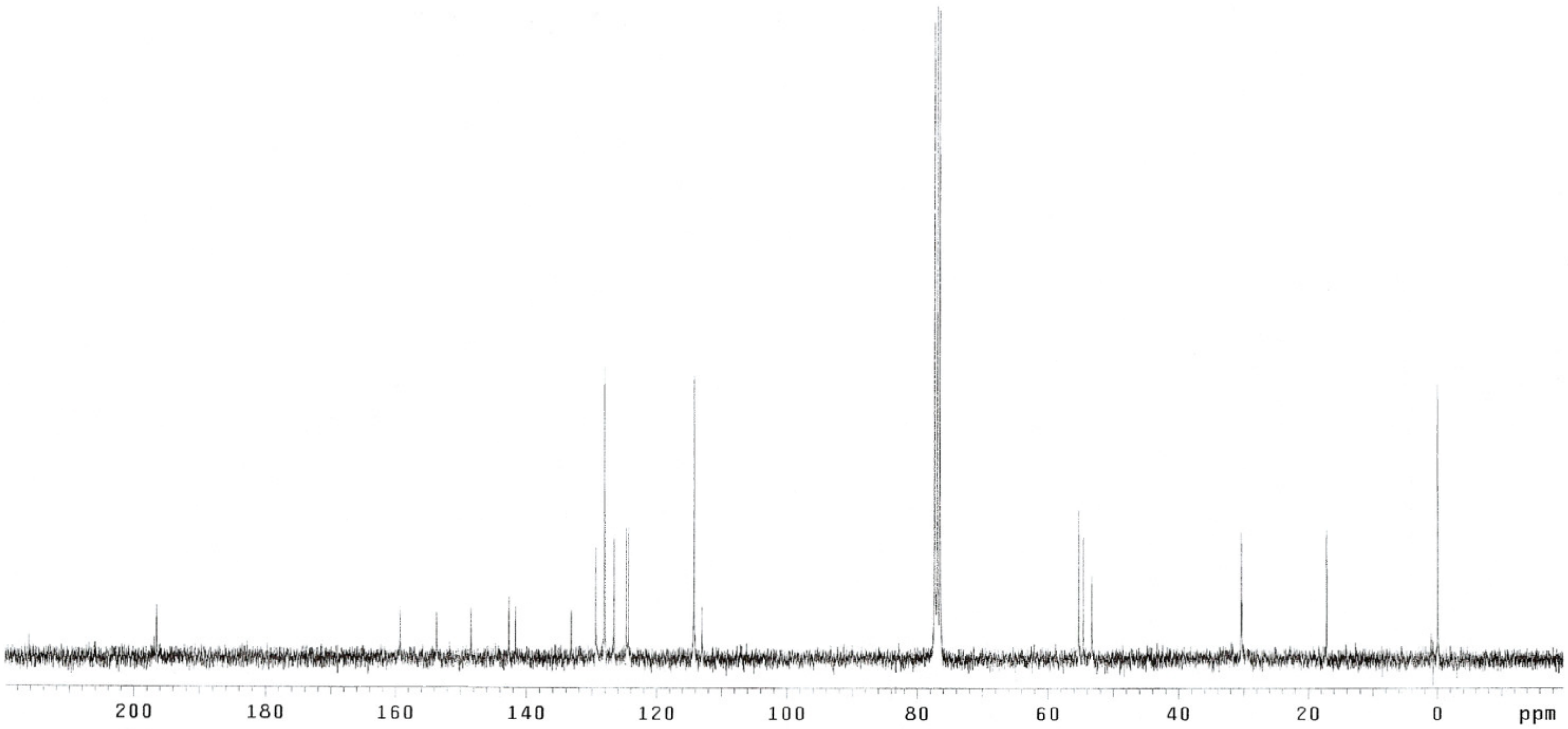

```
ple Name : A5B1C5 Cycl
=================================================================
. Operator    : TRINH                          Seq. Line :    2
. Instrument  : Instrument 1                    Location : Vial 2
ection Date   : 5/25/2011 12:15:17 PM                Inj :    1
                                              Inj Volume : 4 µl
hod           : C:\CHEM32\1\METHODS\GEMETHOD1.M
t changed     : 4/27/2011 11:04:42 AM by J
```

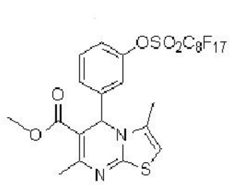

8b

Current Chromatogram(s)

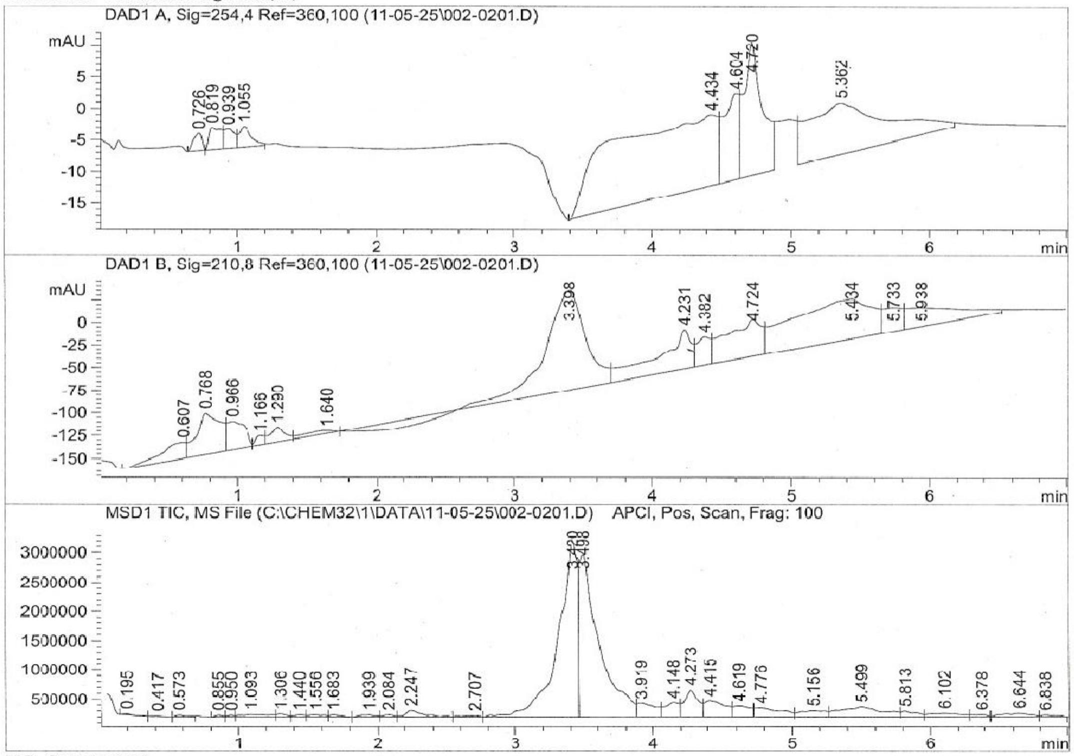

MS Spectrum

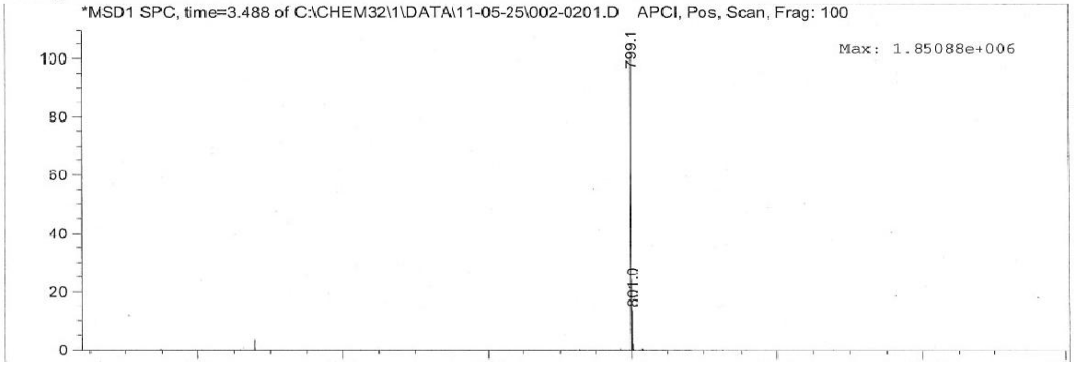

STANDARD 1H OBSERVE

Pulse Sequence: s2pul
Solvent: CDCl3
Ambient temperature
GEMINI-300BB "Mighty300"

Relax. delay 1.000 sec
Pulse 43.4 degrees
Acq. time 1.998 sec
Width 4500.5 Hz
32 repetitions
OBSERVE H1, 300.1266235 MHz
DATA PROCESSING
FT size 32768
Total time 1 min, 39 sec

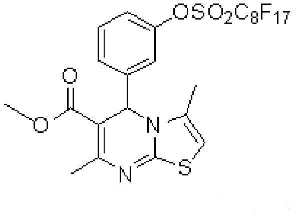

8b

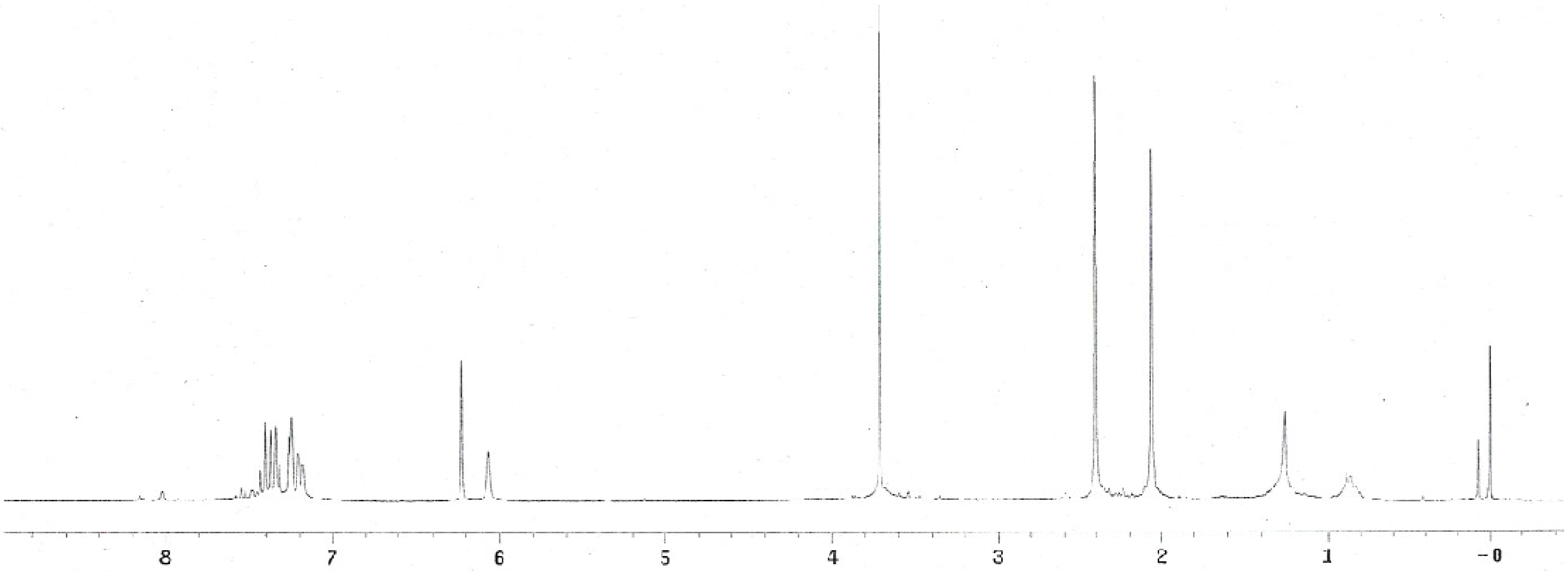

13C OBSERVE

Pulse Sequence: s2pul
Solvent: CDCl3
Ambient temperature
GEMINI-300BB "Mighty300"

Pulse 67.8 degrees
Acq. time 1.815 sec
Width 18761.7 Hz
1024 repetitions
OBSERVE C13, 75.4669007 MHz
DECOUPLE H1, 300.1281260 MHz
Power 36 dB
continuously on
WALTZ-16 modulated
DATA PROCESSING
Line broadening 1.0 Hz
FT size 131072
Total time 36 min, 3 sec

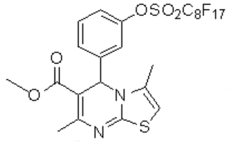

8b

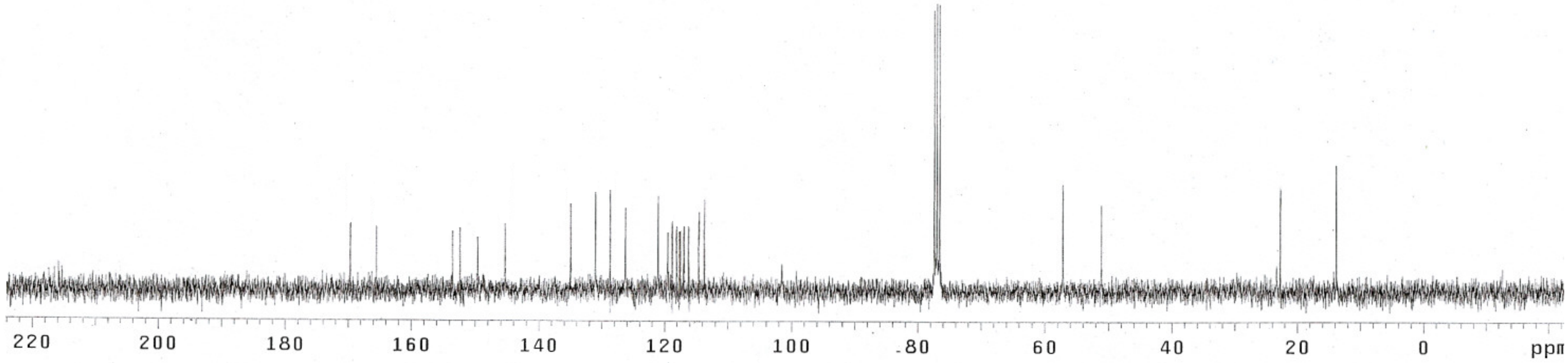

Print of all graphic windows
Data File : C:\CHEM32\1\DATA\11-01-05\008-0501.D
Sample Name : a6b1c5d1 cycload

===

Acq. Operator	: H	Seq. Line :	5
Acq. Instrument	: Instrument 1	Location :	Vial 8
Injection Date	: 1/5/2011 5:22:16 PM	Inj :	1
		Inj Volume :	4 µl
Method	: C:\CHEM32\1\METHODS\GEMETHOD1.M		
Last changed	: 12/23/2010 11:36:52 AM by Z		

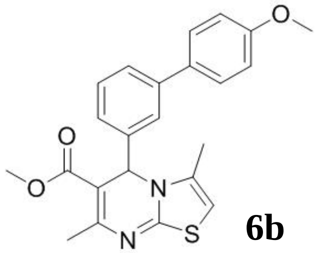

6b

Current Chromatogram(s)

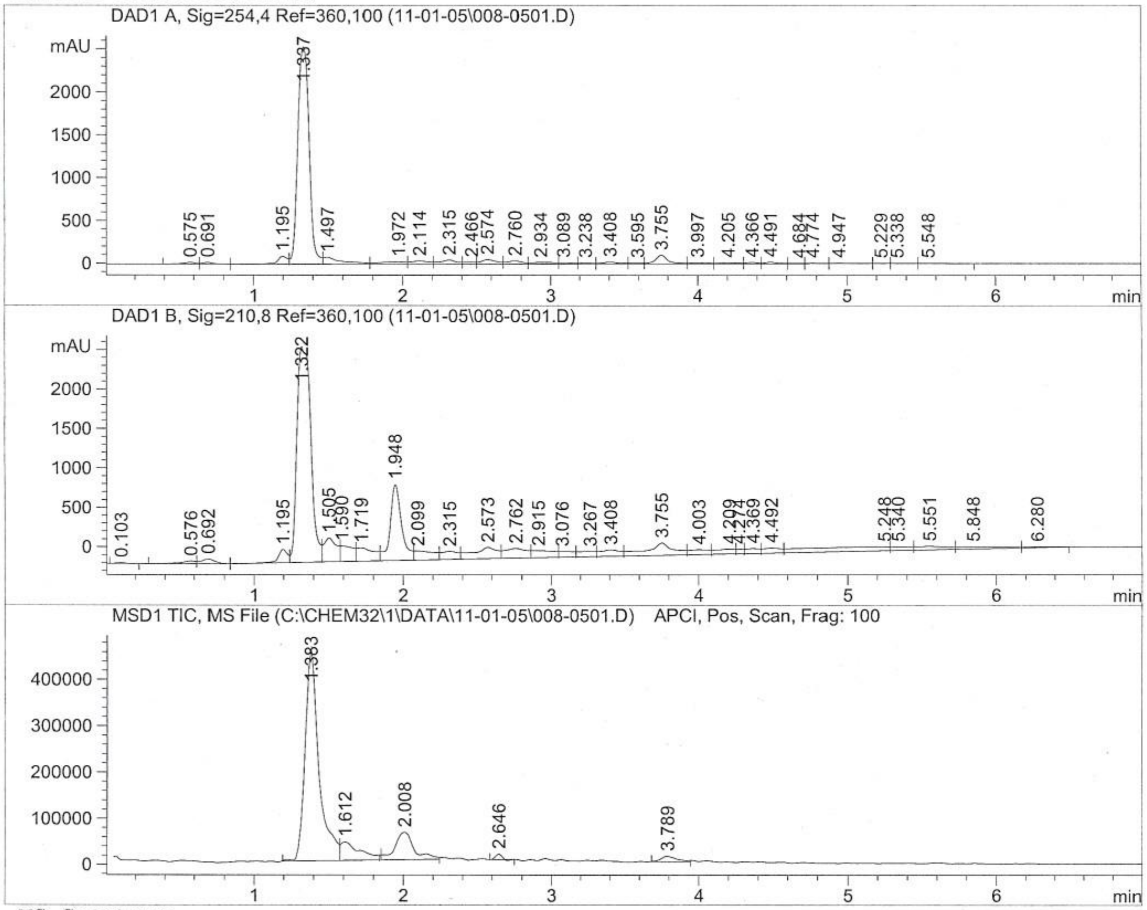

MS Spectrum

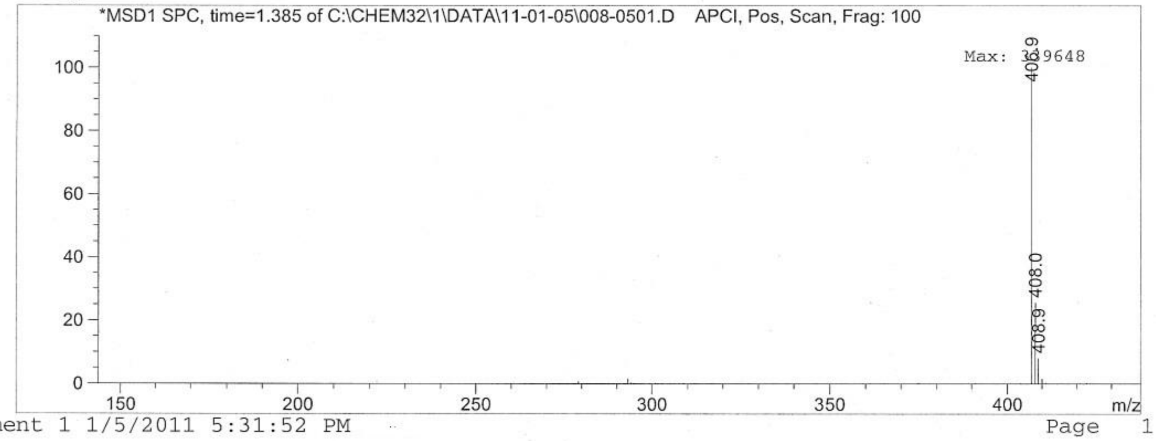

Instrument 1 1/5/2011 5:31:52 PM Page 1 of

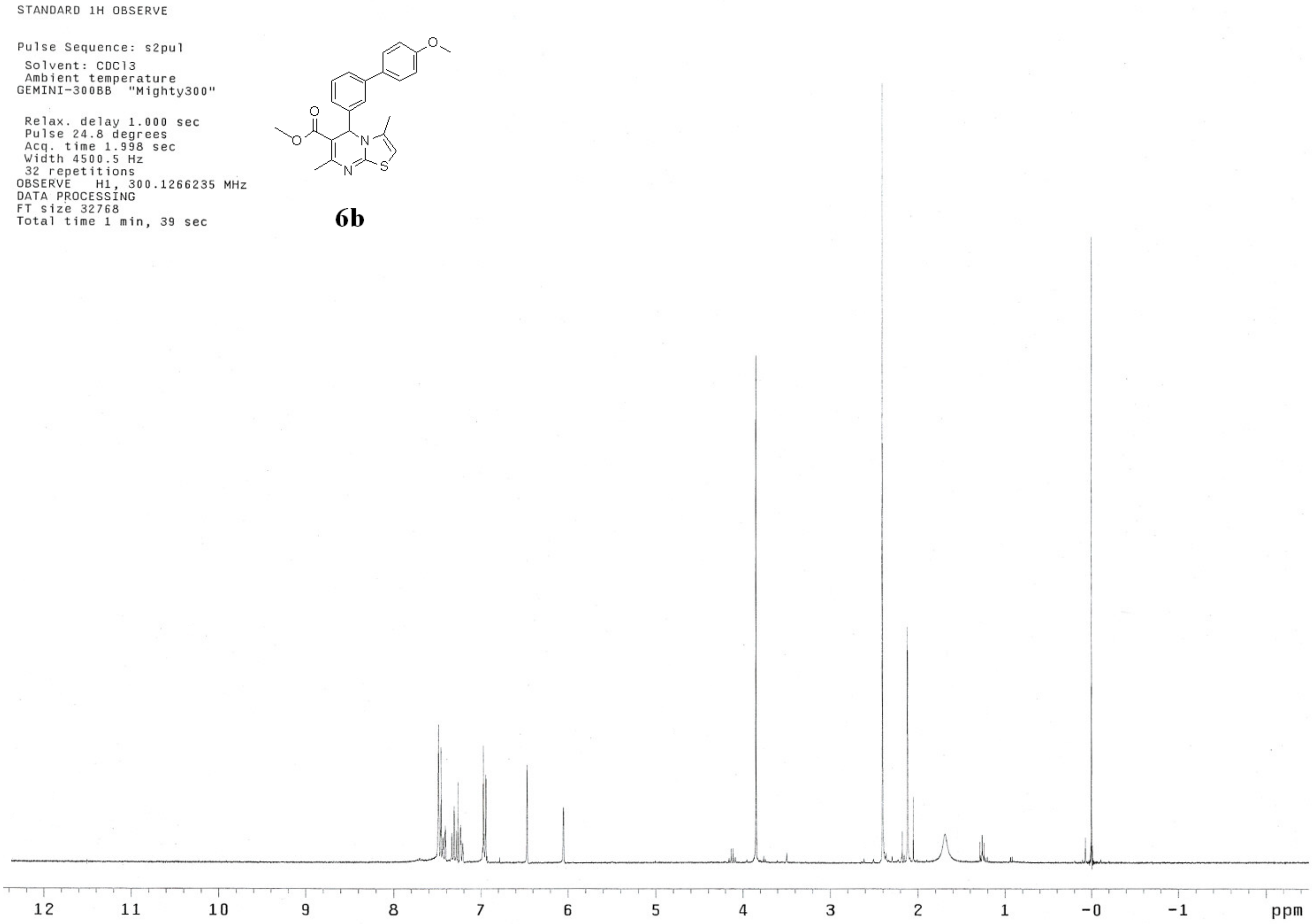
STANDARD 1H OBSERVE
Pulse Sequence: s2pul
Solvent: CDCl3
Ambient temperature
GEMINI-300BB "Mighty300"
Relax. delay 1.000 sec
Pulse 24.8 degrees
Acq. time 1.998 sec
Width 4500.5 Hz
32 repetitions
OBSERVE H1, 300.1266235 MHz
DATA PROCESSING
FT size 32768
Total time 1 min, 39 sec
12 11 10 9 8 7 6 5 4 3 2 1 -0 -1 ppm
O
O
N
N
S
O

6b

13C OBSERVE

Pulse Sequence: s2pul
Solvent: CDCl3
Ambient temperature
GEMINI-300BB "Mighty300"

Pulse 75.9 degrees
Acq. time 1.815 sec
Width 18761.7 Hz
2048 repetitions
OBSERVE C13, 75.4669015 MHz
DECOUPLE H1, 300.1281260 MHz
Power 36 dB
continuously on
WALTZ-16 modulated
DATA PROCESSING
Line broadening 1.0 Hz
FT size 131072
Total time 1 hr, 12 min, 7 sec

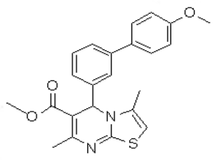

6b

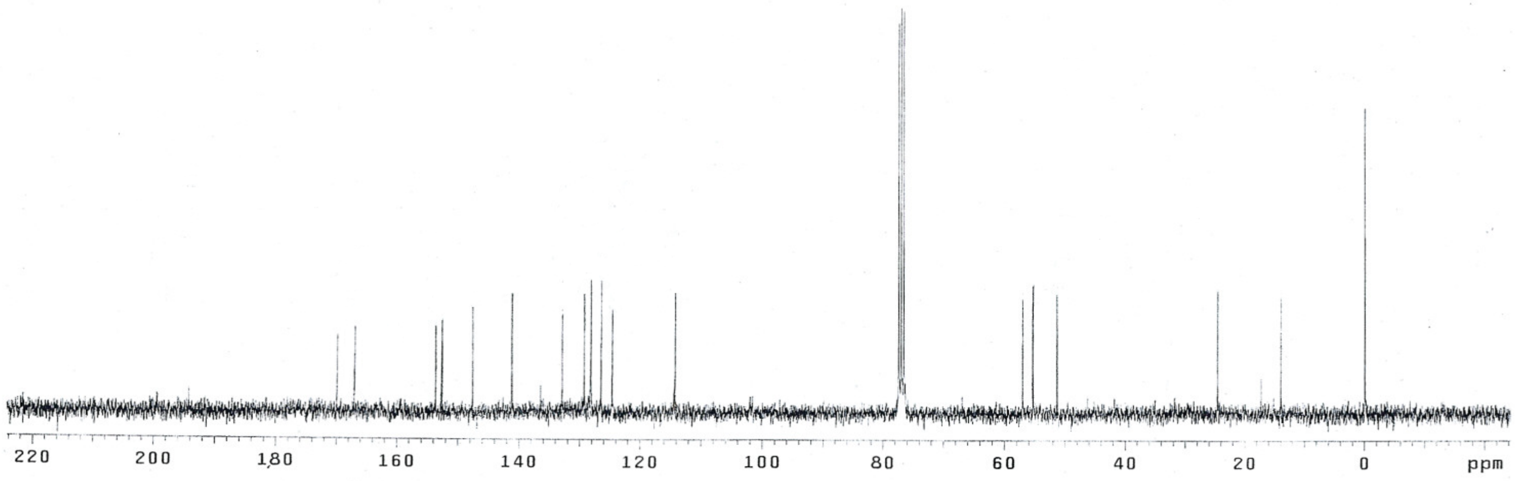

```
Print of all graphic windows
Data File  : C:\CHEM32\1\DATA\11-04-18\008-0401.D
Sample Name : FR-58
=================================================================
Acq. Operator    : Bruno                         Seq. Line :   4
Acq. Instrument : Instrument 1                    Location : Vial 8
Injection Date  : 4/18/2011 4:00:49 PM                 Inj :   1
                                                Inj Volume : 4 µl
Acq. Method     : C:\CHEM32\1\METHODS\GEMETHOD1.M
Last changed    : 3/10/2011 9:35:54 PM by Zijuan
Analysis Method : C:\CHEM32\1\METHODS\CINCHONA.M
Last changed    : 3/18/2011 4:19:30 PM by matt
```

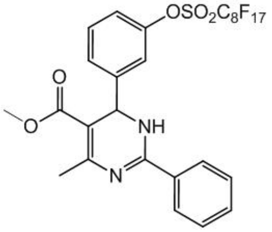

9

Current Chromatogram(s)

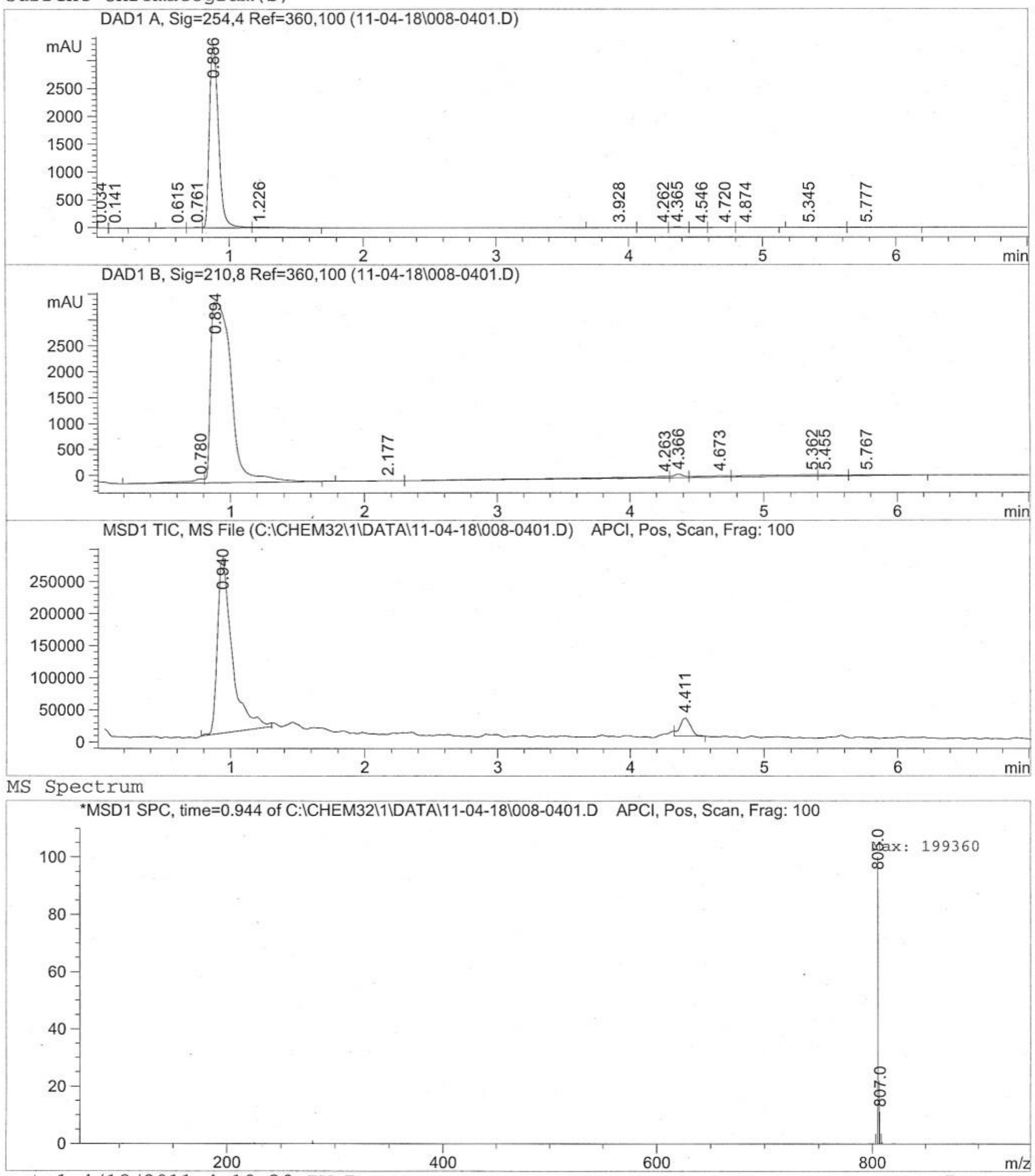

Instrument 1 4/18/2011 4:10:30 PM Bruno Page 1 of

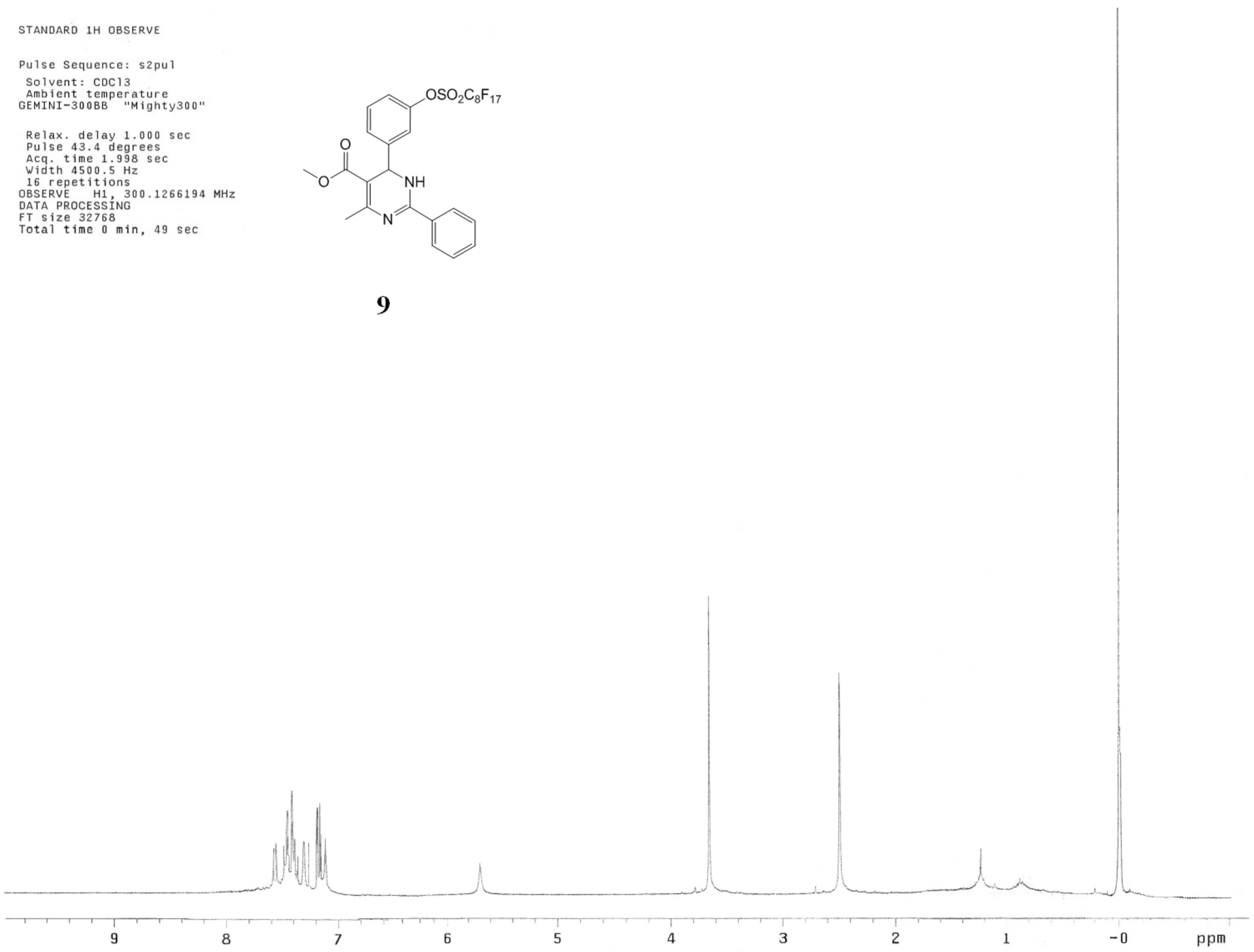
STANDARD 1H OBSERVE
Pulse Sequence: s2pul
Solvent: CDCl3
Ambient temperature
GEMINI-300BB "Mighty300"
Relax. delay 1.000 sec
Pulse 43.4 degrees
Acq. time 1.998 sec
Width 4500.5 Hz
16 repetitions
OBSERVE H1, 300.1266194 MHz
DATA PROCESSING
FT size 32768
Total time 0 min, 49 sec
OSO2C8F17
O
O
NH
N
9
9
8
7
6
5
4
3
2
1
-0
ppm

13C OBSERVE

Pulse Sequence: s2pul
Solvent: CDCl3
Ambient temperature
GEMINI-300BB "Mighty300"

Pulse 75.9 degrees
Acq. time 1.815 sec
Width 18761.7 Hz
3072 repetitions
OBSERVE C13, 75.4669010 MHz
DECOUPLE H1, 300.1281260 MHz
Power 36 dB
continuously on
WALTZ-16 modulated
DATA PROCESSING
Line broadening 1.0 Hz
FT size 131072
Total time 1 hr, 48 min, 10 sec

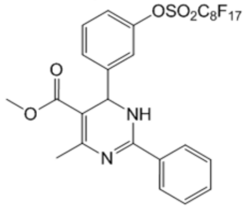

9

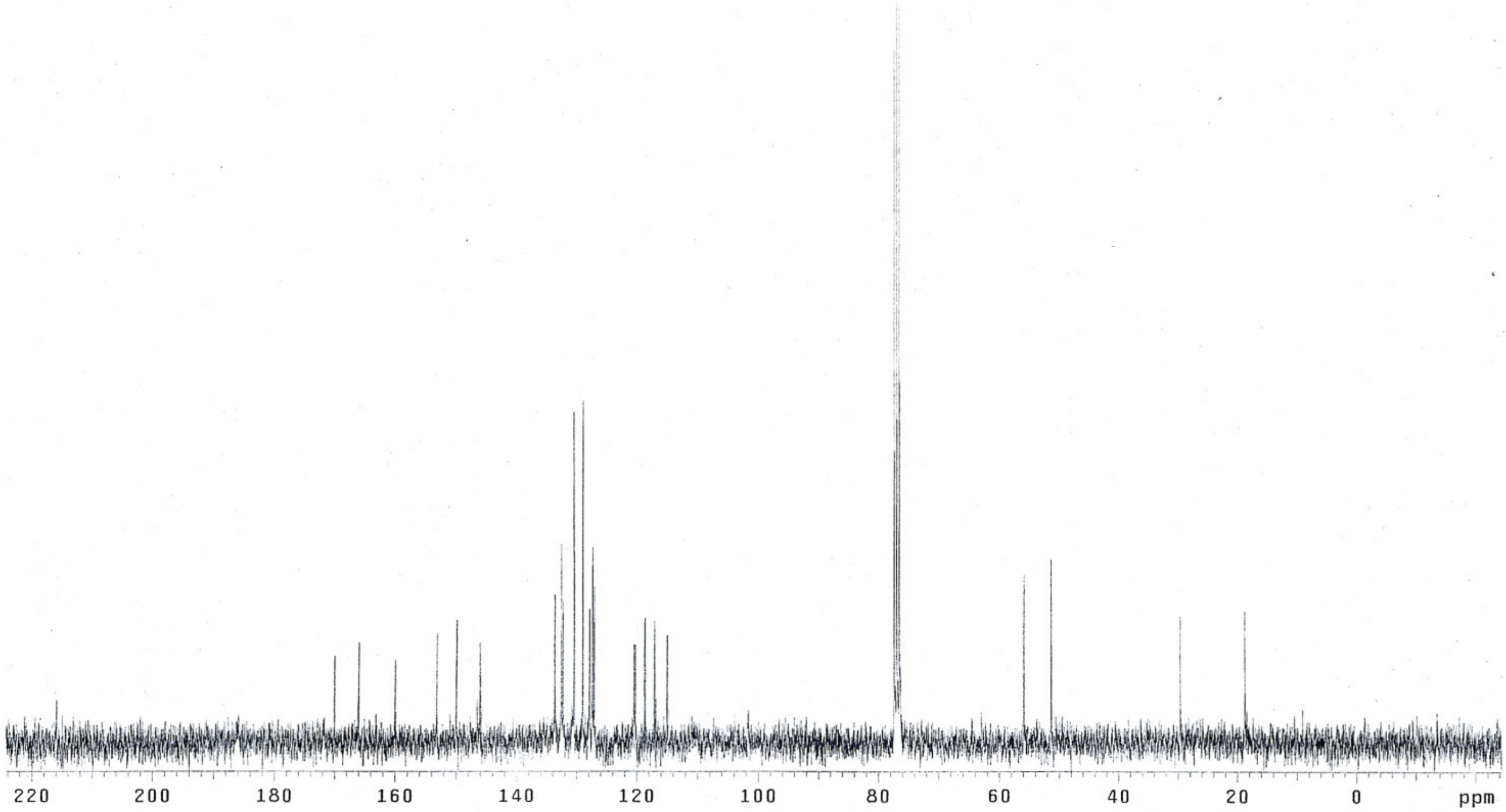

```
Print of all graphic windows
Data File  : C:\CHEM32\1\DATA\11-01-04\SIG1000001.D
Sample Name : Suz Lib-S 412
=====================================================================
Acq. Operator   : Bruno                          Seq. Line :   1
Acq. Instrument : Instrument 1                    Location : Vial 5
Injection Date  : 1/4/2011 2:22:04 PM                   Inj :   1
                                               Inj Volume : 4 µl
Acq. Method     : C:\CHEM32\1\METHODS\GEMETHOD1.M
Last changed    : 12/23/2010 11:36:52 AM by Zijuan
Analysis Method : C:\CHEM32\1\METHODS\CINCHONA.M
Last changed    : 9/23/2010 6:12:44 PM by Stephanie
```

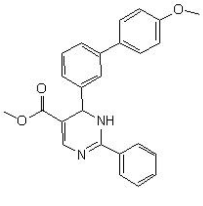

7b

Current Chromatogram(s)

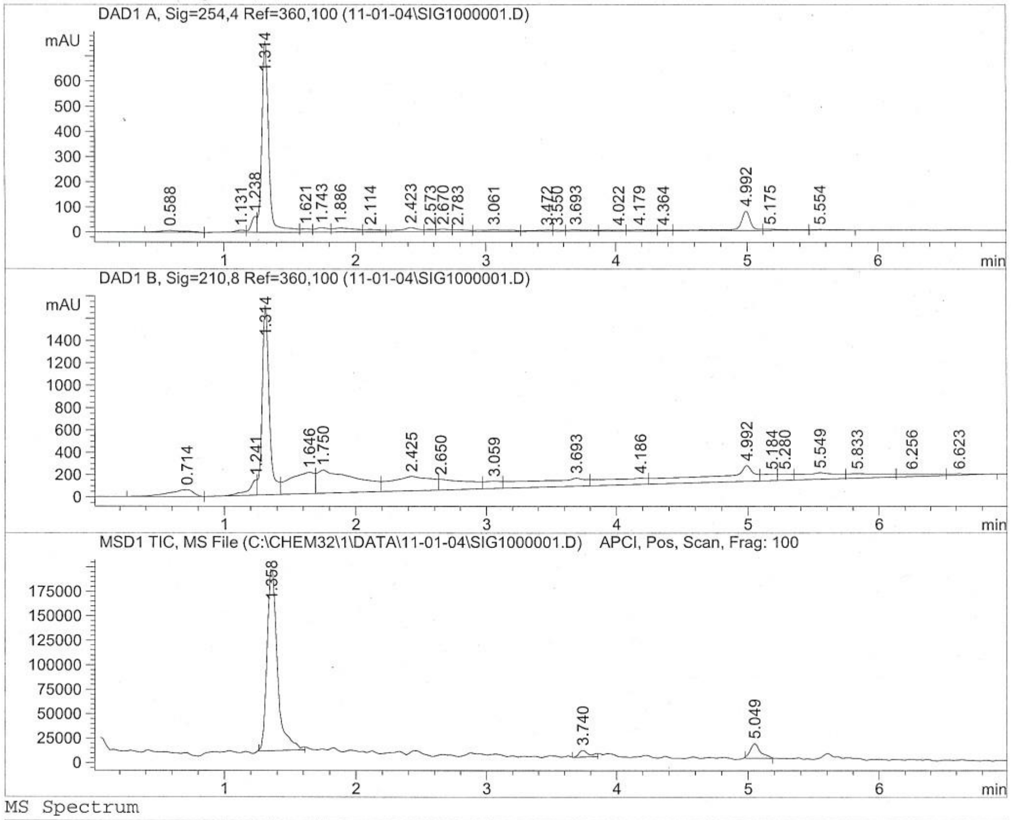

MS Spectrum

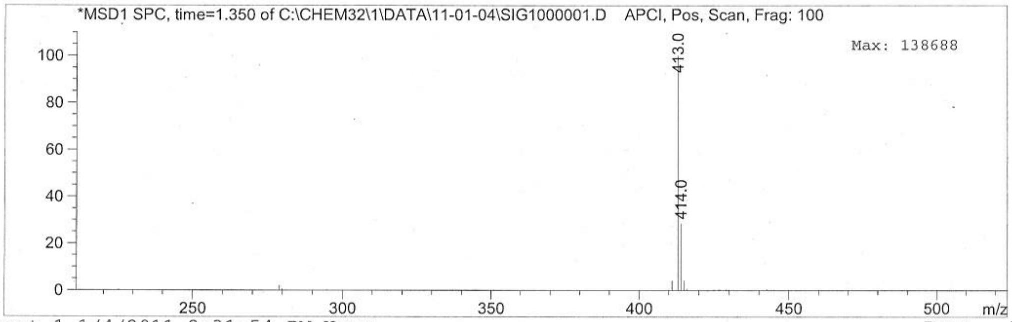

Instrument 1 1/4/2011 2:31:54 PM Hong   Page 1 of 1

STANDARD 1H OBSERVE

Pulse Sequence: s2pul
Solvent: CDCl3
Ambient temperature
GEMINI-300BB "Mighty300"

Relax. delay 1.000 sec
Pulse 43.4 degrees
Acq. time 1.998 sec
Width 4500.5 Hz
64 repetitions
OBSERVE H1, 300.1266246 MHz
DATA PROCESSING
FT size 32768
Total time 3 min, 19 sec

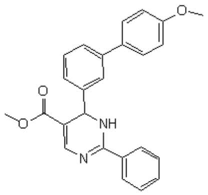

7b

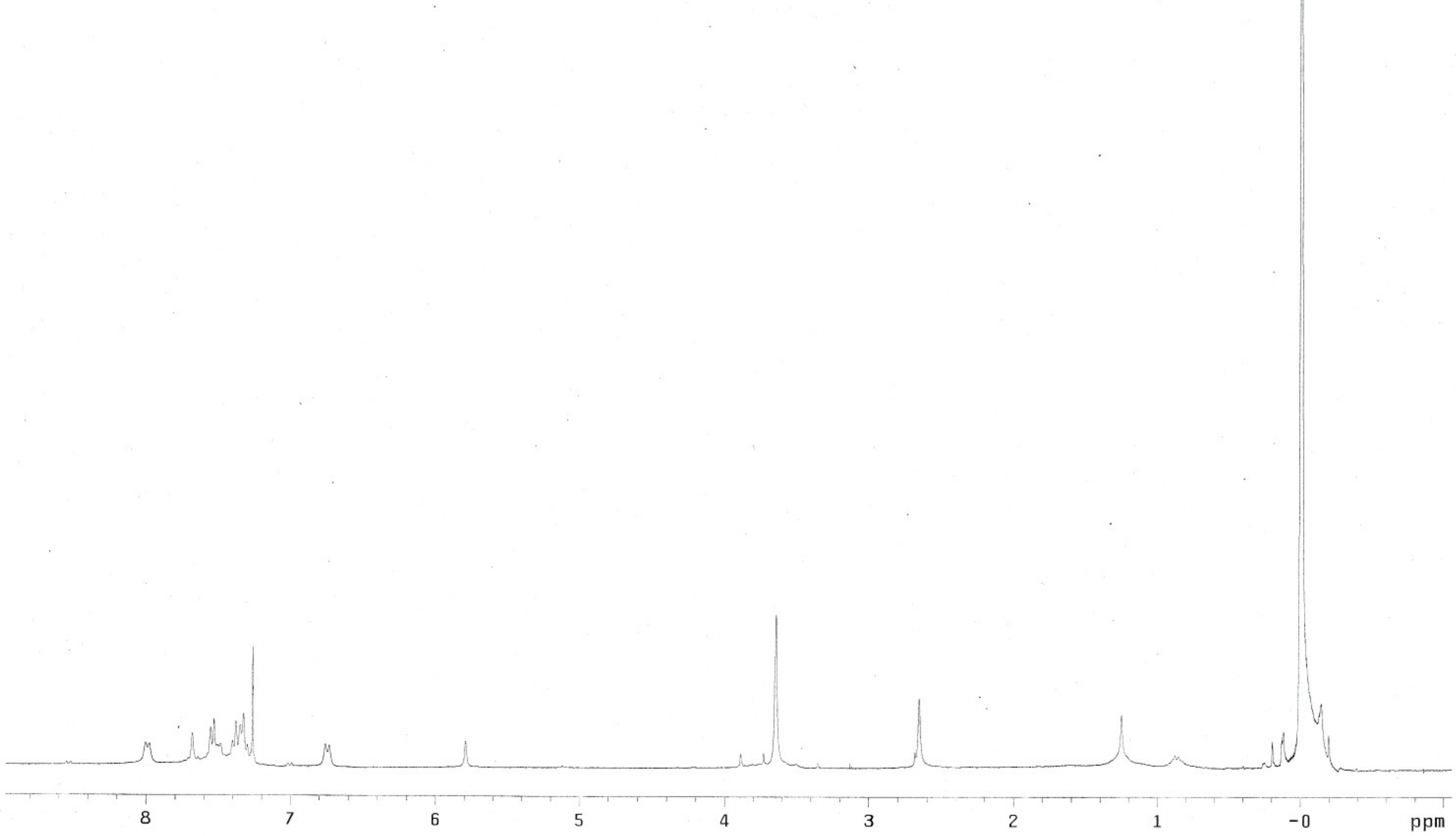

13C OBSERVE

Pulse Sequence: s2pul
Solvent: CDCl3
Ambient temperature
GEMINI-300BB "Mighty300"

Relax. delay 2.000 sec
Pulse 67.8 degrees
Acq. time 1.815 sec
Width 20000.0 Hz
1530 repetitions
OBSERVE C13, 75.4669000 MHz
DECOUPLE H1, 300.1281260 MHz
Power 36 dB
continuously on
WALTZ-16 modulated
DATA PROCESSING
Line broadening 1.0 Hz
FT size 131072
Total time 1 hr, 45 min, 17 sec

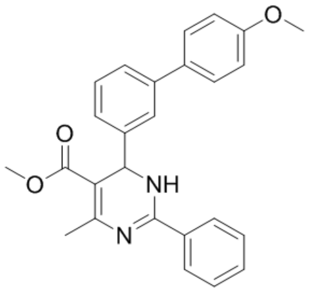

7b

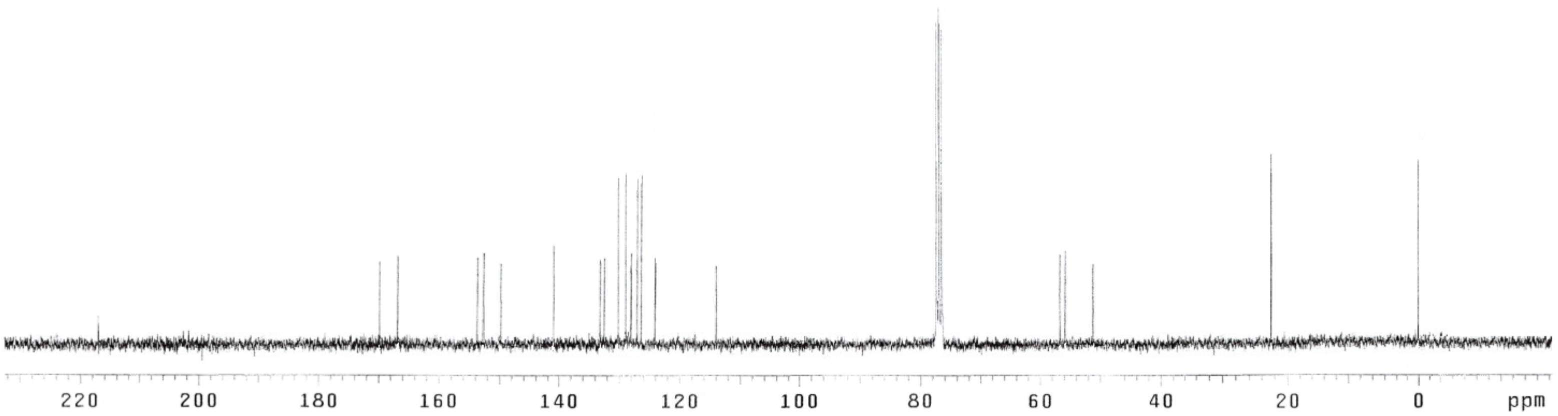

Supplement: File 1 — LC–MS, 1H NMR and 13C NMR data and spectra for compounds 4c, 5a, 6b, 7b, 8b, 9. [file Beilstein_J_Org_Chem-07-1294-s001.pdf]
